# The risk of Alzheimer’s disease according to dynamic changes in metabolic health and obesity: a nationwide population-based cohort study

**DOI:** 10.18632/aging.203255

**Published:** 2021-07-08

**Authors:** Yun Kyung Cho, Jiwoo Lee, Hwi Seung Kim, Joong-Yeol Park, Woo Je Lee, Ye-Jee Kim, Chang Hee Jung

**Affiliations:** 1 Department of Internal Medicine, Hallym University Sacred Heart Hospital, Hallym University College of Medicine, Anyang, Republic of Korea; 2 Department of Internal Medicine, Asan Medical Center, University of Ulsan College of Medicine, Seoul, Republic of Korea; 3 Department of Clinical Epidemiology and Biostatistics, Asan Medical Center, University of Ulsan College of Medicine, Seoul, Republic of Korea

**Keywords:** obesity, metabolic health, Alzheimer’s disease, nationwide cohort study

## Abstract

We evaluated the association of metabolic health and obesity phenotypes with the risk of Alzheimer’s disease (AD).

This study enrolled 136,847 elderly participants aged 60 or above from the Korean National Health Insurance System. At baseline examinations in 2009 and 2010, subjects were categorized into four groups: the metabolically healthy non-obese (MHNO), metabolically healthy obese (MHO), metabolically unhealthy non-obese (MUNO), and metabolically unhealthy obese (MUO) groups. Based on the phenotypic transition after 2 years, the subjects were further categorized into 16 subgroups. They were followed from 2009 to 2015 to monitor for AD development.

The MHO phenotype protected subjects from AD, relative to the MHNO phenotype (HR, 0.73; 95% CI, 0.65–0.81). Among subjects initially classified as MHO, 41.8% remained MHO, with a significantly lower risk of AD compared with the stable MHNO group (HR, 0.62; 95% CI, 0.50–0.77). Among MUO subjects at baseline, those who changed phenotype to MUNO were at higher risk of AD (HR, 1.47; 95% CI, 1.28–1.70), and the transition to the MHO phenotype protected subjects from AD (HR, 0.62; 95% CI, 0.50–0.78).

The MHO phenotype conferred a decreased risk of AD. Maintenance or recovery of metabolic health might mitigate AD risk among obese individuals.

## INTRODUCTION

The global prevalence of dementia has greatly increased and became a major public health issue [1]. The estimated number of patients with dementia is projected to reach 106 million worldwide by 2050 [1]. Alzheimer’s disease (AD) is the most common type of dementia, with an incidence of 60% to 80% [2], but no preventive or disease-modifying therapies are currently available [1]. Hence, the detection of modifiable risk factors is of critical importance.

Obesity is a well-known risk factor for several cardiometabolic diseases including diabetes and cardiovascular disease, and certain types of cancer; recently, obesity has also emerged as an independent risk factor for AD and other forms of dementia [3–6]. Nevertheless, the relationship between the risk of AD and obesity or metabolic disturbances caused by obesity remains controversial, with inconsistent findings across studies [5, 7–11]. Some epidemiologic studies have suggested that obesity has a role in the development of AD, and this observation could be via several pathological alterations in obesity and insulin resistance, such as chronic inflammation and mitochondrial dysfunction [5, 7]. However, more recent studies have identified obesity as protective against dementia, which contradicts previous associations between obesity and AD [6, 8–11]. Furthermore, previously reported associations between obesity and AD should be carefully interpreted, as states of metabolic unhealthiness, such as hyperglycemia and dyslipidemia, are commonly combined with obesity and would confound the effect of obesity itself on the risk of AD [12].

“Metabolically healthy obese” (MHO) individuals are a subpopulation of obese people with a low burden of overt cardiometabolic abnormalities [13–16]. Several studies have reported that MHO people are neither at increased risk of cardiometabolic disease nor mortality compared with normal-weight controls [17, 18]. The prognostic value of MHO, however, could be largely dependent on health outcomes; indeed, a few recent studies have reported that MHO individuals are at even lower risk of AD than previously thought [2, 19].

However, in the obese population, metabolic health status is a modifiable condition. For instance, in the Multi-Ethnic Study of Atherosclerosis (MESA), nearly half of the participants with MHO at baseline progressed to metabolically unhealthy during the approximately 12-year follow-up period [20]. Another prospective cohort research with a 4-year follow-up found that 14.5% of the people with MHO at initial examination progressed to metabolically unhealthy obesity (MUO), whereas 29.0% of those with MUO at baseline recovered their metabolic health [21]. Although the risk of cardiometabolic complications could be influenced by these phenotypic transitions [20, 22, 23], there have been no data generated regarding the risk of AD according to dynamic changes of obesity and metabolic health status.

Therefore, we designed this study to clarify the implication of obesity and metabolic health on AD risk, taking into account phenotypical changes in obese metabolic health status, using a large-scale population dataset from a national health screening examination.

## RESULTS

### Clinical and biochemical characteristics of the entire cohort at baseline examination

**Figure 1:**
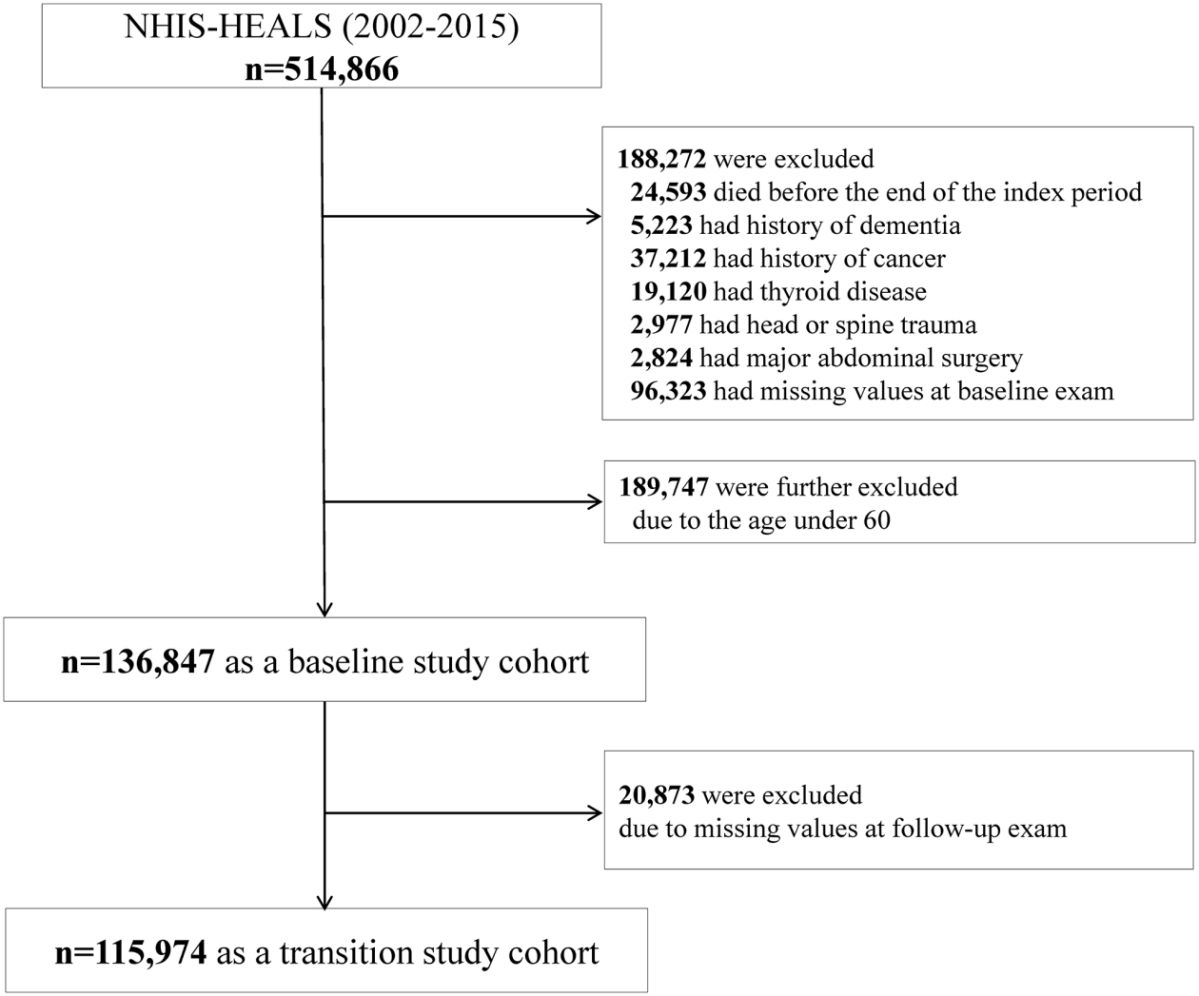
Study enrollment flowchart.

Our baseline study cohort included 136,847 people aged 60 or above (Figure 1). Table 1 shows the baseline characteristics of the participants categorized by the presence of obesity and the metabolic health status. The prevalences of MHNO, MHO, MUNO, and MUO individuals were 24.1% (*n* = 33,049), 7.6% (*n* = 10,445), 39.4% (*n* = 53,958), and 28.8% (*n* = 39,395), respectively. MHO subjects had higher levels of TG, LDL-C, and TC, but lower level of HDL-C, as compared to the referent lean and healthy individuals. MHO subjects, on the other hand, had better lipid profiles than MUNO or MUO subjects. More male subjects were included in the metabolically unhealthy groups (MUNO and MUO).

**Table 1:** Characteristics of study participants according to baseline metabolic health and obesity status.

**Baseline category BMI Metabolic health status**	**MHNO <25 kg/m^2^ 0–1 risk factors**	**MHO ≥25 kg/m^2^ 0–1 risk factors**	**MUNO <25 kg/m^2^ ≥2 risk factors**	**MUO ≥25 kg/m^2^ ≥2 risk factors**	***P* value**
***n***	33, 049 (24.1)	10, 445 (7.6)	53, 958 (39.4)	39, 395 (28.8)	
**Sex (% men)**	55.0	45.2	49.3	45.5	<.0001
**Age (years)**	67.3 ± 6.0^a^	66.4 ± 5.4^a^	68.0 ± 6.0	67.1 ± 5.5	<.0001
**BMI (kg/m^2^)**	21.9 ± 2.0	26.8 ± 1.7	22.6 ± 1.8	27.2 ± 1.9	<.0001
**WC (cm)**	89.2 ± 6.8	87.8 ± 6.4	80.8 ± 6.5	89.7 ± 6.6	<.0001
**Systolic BP (mmHg)**	122.6 ± 15.1	126.0 ± 14.8	131.3 ± 15.3	133.5 ± 15.0	<.0001
**Diastolic BP (mmHg)**	74.9 ± 9.6	76.9 ± 9.6	79.5 ± 9.7	80.9 ± 9.7	<.0001
**Smoking (%)**					<.0001
** Smoker**	15.0	8.8	14.3	9.9	
** Non-smoker**	85.0	91.2	85.7	90.1	
**Drinking (%)**					<.0001
** Non-heavy**	87.4	88.3	86.5	86.7	
** Heavy**	12.6	11.7	13.5	13.3	
**Physical activity (%)**					<.0001
** None**	30.1	30.2	31.5	32.3	
** 1–4 times/week**	36.2	36.7	35.9	37.2	
** ≥5 times/week**	33.7	33.1	32.5	30.6	
**Medical history (%)**					
** Type 2 diabetes**	1.8	1.6	20.7	23.8	<.0001
** HTN**	23.2	35.4	59.5	70.6	<.0001
** Dyslipidemia**	4.3	4.3	35.8	42.2	<.0001
**Income**					
** Medical aid**	0.3	0.4	0.4	0.5	<.0001
** Low**	37.4	36.8	38.5	38.4	
** High**	62.2	62.9	61.1	61.1	
**FPG (mg/dl)**	91.7 ± 13.2	92.0 ± 11.9	108.3 ± 29.1	109.9 ± 28.7	<.0001
**TG (mg/dl)**	97.6 ± 42.9	106.0 ± 41.7	157.4 ± 93.3	171.9 ± 95.7	<.0001
**LDL-C (mg/dl)**	59.6 ± 28.2	58.0 ± 27.2	52.1 ± 29.0	50.3 ± 24.8	<.0001
**HDL-C (mg/dl)**	118.7 ± 33.9	124.8 ± 32.7	118.8 ± 40.1	120.2 ± 40.2	<.0001
**TC (mg/dl)**	196.6 ± 33.4	202.9 ± 33.5^a^	200.6 ± 40.4^a^	203.2 ± 40.7	<.0001

Results reported as means ± SD, unless otherwise indicated.

All variables were statistically different among the 4 groups, unless otherwise indicated.

^a^No statistical difference was observed.

Abbreviations: BMI: body mass index; BP: blood pressure; eGFR: estimated glomerular filtration rate; FPG: fasting plasma glucose; HDL-C: high-density lipoprotein cholesterol; HTN: hypertension; IBD: inflammatory bowel disease; LDL-C: low-density lipoprotein cholesterol; MHO: stable metabolically healthy obesity; MUO: metabolically unhealthy obesity; MUNO: metabolically unhealthy obesity; MHNO: metabolically healthy non-obesity; TC: total cholesterol; TG: triglyceride; WC: waist circumference.

### The risk of AD according to baseline metabolic health and obesity status

During the follow-up period (median, 71.5 months; interquartile ranges, 66.0–78.5), 7,043 of the 136,847 individuals (5.1%) developed AD. The crude incidence rate of AD was 4.7% (1,540/33,049) in the MHNO group, 3.6% (374/10,445) in the MHO group, 6.1% (3,272/53,958) in the MUNO group, and 4.7% (1,857/39,395) in the MUO group.

Table 2 demonstrates the risk of AD development according to the metabolic health and obesity status at baseline, without considering phenotypic transitions over time. Multivariable-adjusted HRs (95% CIs) of the MHO, MUNO, and MUO groups for AD were 0.73 (0.65–0.81), 1.28 (1.21–1.36), and 0.96 (0.90–1.03), respectively, relative to the MHNO group after adjusting for confounders (Table 2). Subjects in the MHO group had a significantly lower HR for AD, and MUNO subjects had a higher risk of developing dementia risk than the MHNO group.

**Table 2:** Risk of AD according to baseline metabolic health and obesity status.

**Baseline category BMI Metabolic health status**	**MHNO <25 kg/m^2^ 0–1 risk factors**	**MHO ≥25 kg/m^2^ 0–1 risk factors**	**MUNO <25 kg/m^2^ ≥2 risk factors**	**MUO ≥25 kg/m^2^ ≥2 risk factors**
*n* (% of total)	17, 812 (24.2)	10, 445 (7.6)	53, 958 (39.4)	39, 395 (28.8)
Number of events (%)	1, 540 (4.66)	374 (3.58)	3, 272 (6.06)	1, 857 (4.71)
Crude HR (95% CI)	1 (ref)	0.76 (0.68–0.85)	1.32 (1.24–1.40)	1.01 (0.94–1.08)
Multivariable-adjusted HR (95% CI)^a^	1 (ref)	0.73 (0.65–0.81)	1.28 (1.21–1.36)	0.96 (0.90–1.03)

^a^Adjusted for baseline age, sex, income, smoking, alcohol drinking, and physical activities.

Abbreviations: MHO: stable metabolically healthy obesity; MUO: metabolically unhealthy obesity; MUNO; metabolically unhealthy obesity; MHNO: metabolically healthy non-obesity.

### Changes in metabolic phenotype at follow-up examinations

**Figure 2:**
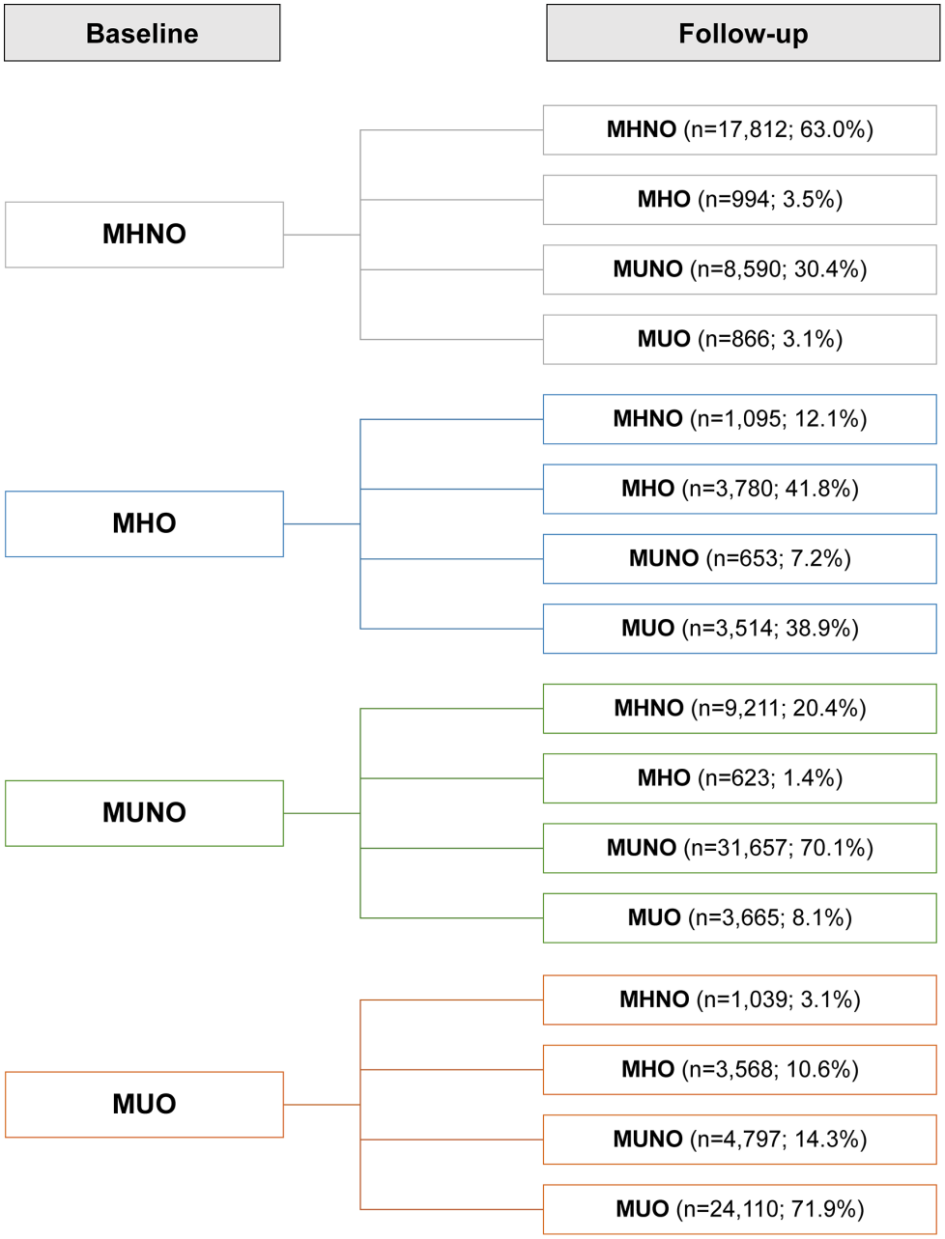
**Prevalence of phenotypic transitions at follow-up visits.** Abbreviations: MHO: stable metabolically healthy obesity; MUO: metabolically unhealthy obesity; MUNO: metabolically unhealthy obesity; MHNO: metabolically healthy non-obesity.

We investigated how obese metabolic health status would change over the follow-up period (Figure 2). Overall, 33.3% of the subjects experienced phenotypic changes during follow-up. In the baseline MHNO, MUNO, and MUO groups, more than half of the subjects maintained their baseline phenotypes at their follow-up visits, while 41.8% of the MHO individuals experienced no phenotype changes (the stable MHO group). In the MHNO group, 33.5% of the subjects were metabolically unhealthy at the end of follow-up: 30.4% became MUNO, and 3.1% became MUO; 3.5% transitioned to the MHO group. Among the individuals who were MUO at baseline, 10.6% recovered their metabolic health without changing their obesity status (i.e., transitioned from the MUO to MHO group).

### The risk of AD according to phenotypic transitions in metabolic health and obesity status

**Figure 3:**
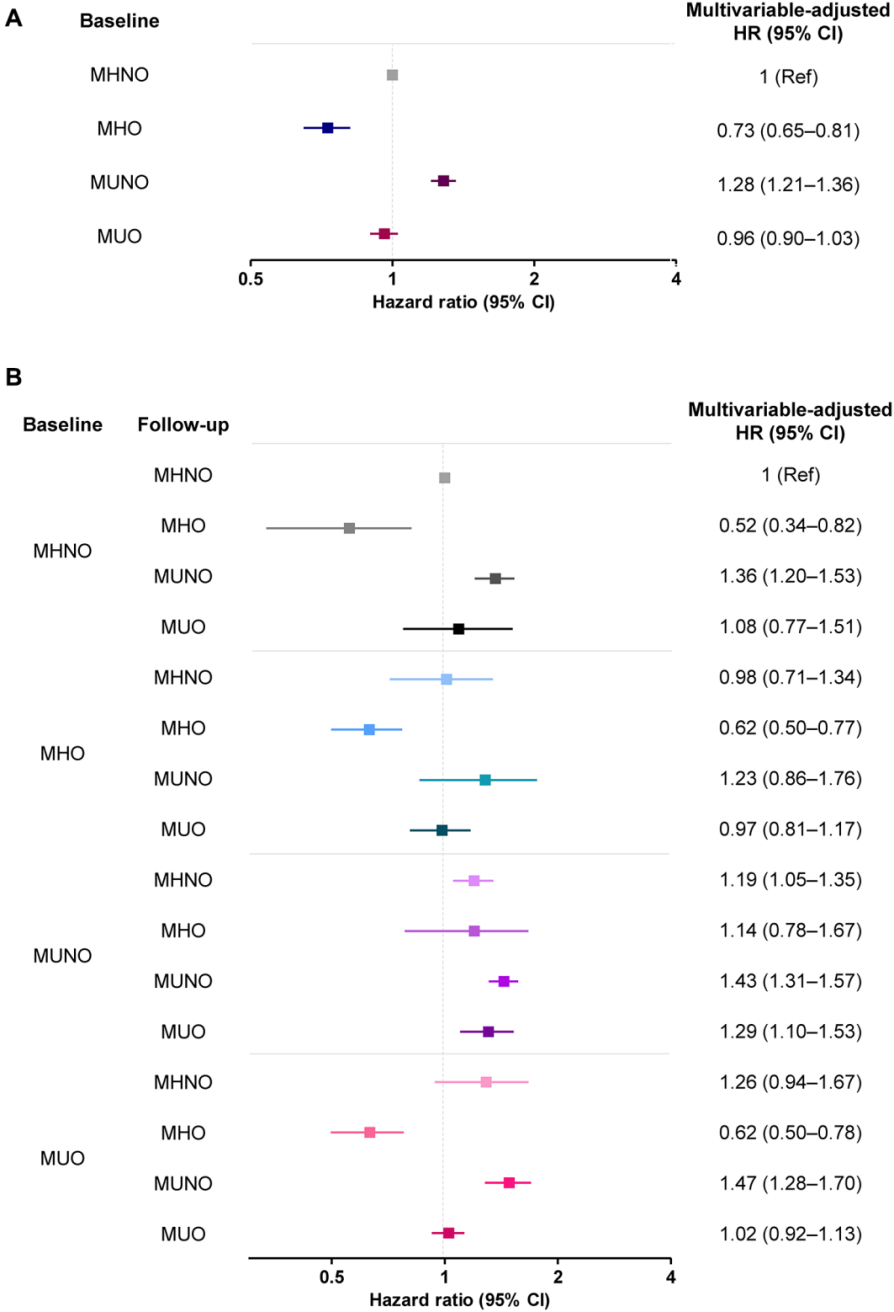
The risk of AD (**A**) without and (**B**) with consideration of transition in body mass and metabolic health phenotypes. Abbreviations: MHO: stable metabolically healthy obesity; MUO: metabolically unhealthy obesity; MUNO: metabolically unhealthy obesity; MHNO: metabolically healthy non-obesity.

Table 3 and Figure 3 show the multivariable-adjusted HRs for AD among the 16 subgroups according to their metabolic and obese phenotypes considering the transitions, using the stable MHNO group as the reference. Compared with the stable MHNO group, the stable MHO group had a significantly lower risk of AD (multivariable-adjusted HR, 0.62; 95% CI, 0.50–0.77). The MHO subjects who experienced phenotypic transitions to any other phenotype were not significantly different from the reference group in terms of AD risk.

**Table 3:** Risk of AD according to transitions of metabolic health and obesity status.

**Baseline category**	**MHNO**			
**Follow-up category**	**MHNO**	**MHO**	**MUNO**	**MUO**
***n* (% of baseline category)**	17, 812 (63.0)	994 (3.5)	8, 590 (30.4)	866 (3.1)
**Number of events (%)**	661 (3.71)	20 (2.01)	434 (5.05)	36 (4.16)
**Crude HR (95% CI)**	1 (ref)	0.52 (0.33–0.81)	1.36 (1.20–1.53)	1.08 (0.77–1.51)
**Multivariable-adjusted HR (95% CI)^a^**	1 (ref)	0.52 (0.34–0.82)	1.36 (1.20–1.53)	1.08 (0.77–1.51)
**Baseline category**	**MHO**
**Follow-up category**	**MHNO**	**MHO**	**MUNO**	**MUO**
***n* (% of baseline category)**	1, 095 (12.1)	3, 780 (41.8)	653 (7.2)	3, 514 (38.9)
**Number of events (%)**	41 (3.74)	92 (2.43)	31 (4.75)	135 (3.84)
**Crude HR (95% CI)**	0.98 (0.71–1.34)	0.62 (0.49–0.77)	1.22 (0.85–1.75)	0.97 (0.81–1.17)
**Multivariable-adjusted HR (95% CI)^a^**	0.98 (0.71–1.34)	0.62 (0.50–0.77)	1.23 (0.86–1.76)	0.97 (0.81–1.17)
**Baseline category**	**MUNO**
**Follow-up category**	**MHNO**	**MHO**	**MUNO**	**MUO**
***N* (% of baseline category)**	9, 211 (20.4)	623 (1.4)	31, 677 (70.1)	3, 665 (8.1)
**Number of events (%)**	414 (4.49)	28 (4.49)	1,714 (5.41)	182 (4.97)
**Crude HR (95% CI)**	1.19 (1.06–1.35)	1.14 (0.78–1.66)	1.43 (1.31–1.56)	1.30 (1.10–1.53)
**Multivariable-adjusted HR (95% CI)^a^**	1.19 (1.05–1.35)	1.14 (0.78–1.67)	1.43 (1.31–1.57)	1.29 (1.10–1.53)
**Baseline category**	**MUO**
**Follow-up category**	**MHNO**	**MHO**	**MUNO**	**MUO**
***n* (% of baseline category)**	1, 039 (3.1)	3.568 (10.6)	4, 797 (14.3)	24, 110 (71.9)
**Number of events (%)**	50 (4.81)	87 (2.44)	275 (5.73)	961 (3.99)
**Crude HR (95% CI)**	1.26 (0.94–1.68)	0.62 (0.50–0.78)	1.47 (1.28–1.70)	1.02 (0.92–1.13)
**Multivariable-adjusted HR (95% CI)^a^**	1.26 (0.94–1.67)	0.62 (0.50–0.78)	1.47 (1.28–1.70)	1.02 (0.92–1.13)

^a^Adjusted for baseline age, sex, income, smoking, alcohol drinking, and physical activities.

Abbreviations: MHO: stable metabolically healthy obesity; MUO: metabolically unhealthy obesity; MUNO: metabolically unhealthy obesity; MHNO: metabolically healthy non-obesity.

In the baseline MUO group, the transition to the MHO phenotype significantly lowered the risk of AD (multivariable-adjusted HR, 0.62; 95% CI, 0.50–0.78), while the transition to MUNO status, or the regression of obesity without the improvement of metabolic health, carried a higher risk of AD (multivariable-adjusted HR, 1.47; 95% CI, 1.28–1.70). Similarly, the transition to a MUNO status from an MHNO status or the persistence of a MUNO status also increased the risk of AD development, relative to the risk borne by individuals in the stable MHNO group (multivariable-adjusted HR, 1.36; 95% CI, 1.20–1.53 and multivariable-adjusted HR, 1.29; 95% CI, 1.10–1.53, respectively, Table 3). The risks of AD without and with consideration of transitions in body mass and metabolic health phenotypes are summarized in Figure 3.

## DISCUSSION

In this nationwide population-based cohort study, we showed that the risk of AD was significantly reduced among MHO individuals, which was in line with the results of previous studies [2, 19]. Additionally, we analyzed the risk of AD according to phenotypic transitions observed during subsequent biannual examinations. The results showed that the protective tendency against AD was maintained among MHO individuals who maintained their MHO status (multivariable-adjusted HR, 0.62; 95% CI, 0.50–0.77), while this tendency disappeared when the obese metabolic phenotype changed. Importantly, the transition to MHO from MUO lowered the risk of AD compared with the maintenance of a stable MHNO status (multivariable-adjusted HR, 0.62; 95% CI, 0.50–0.78), further supporting the protective effect of the MHO phenotype against AD.

Previous investigations on the relationship between obesity and the development of AD have suggested midlife obesity is related with a 1.7- to 2.0-fold increased risk of dementia and AD [24, 25]. On the contrary, most recent studies have shown that being overweight or obese in old age protects against AD [10, 11, 26–28]. However, these previous studies did not consider metabolic unhealthiness, which frequently accompanies obesity, as a factor that potentially contributes to AD risk [12, 29]. A case–control study in a Western population suggested that AD was associated with the metabolic syndrome after adjusting for age, sex, and location [12]. In contrast, Raffaitin et al. investigated the associations of metabolic syndrome and its individual components with AD risk; they found that the presence of metabolic syndrome did not increase the risk of AD [29]. However, upon further analysis, high waist circumference—one of the components of metabolic syndrome—was inversely associated with AD risk (HR,0.63; 95% CI; 0.43–0.94). This inverse relationship suggested a protective effect of obesity, including in terms of possibly mitigating the negative effects of other metabolic abnormalities on AD development.

Recently, two cohort studies investigated the implications of obesity without metabolic unhealthiness on AD incidence [2, 19]. A Korean study using a nationwide cohort reported that the MHO group was at the lowest risk of AD (HR 0.87; 95% CI, 0.86–0.88) relative to the MHNO group [19]. Similarly, in a longitudinal study of 1,199 European individuals (drawn from the Alzheimer’s Disease Neuroimaging Initiative [ADNI] database) who were initially free of AD, the risk of AD among elderly obese individuals was significantly reduced after correcting for metabolic status (HR, 0.70; 95% CI, 0.56–0.89) [2]. Our study results are in line with the results of these previous studies, which demonstrated significantly lower AD risk among MHO subjects (HR, 0.73; 95% CI, 0.65–0.81; Table 2 and Figure 3A).

However, the clinical implications of an MHO status should also be considered in the context of transitory metabolic health states. According to previous reports, one-third to one-half of MHO patients proceeded to a metabolically unhealthy condition, whereas in a quarter to one-third of MUO patients the metabolic fitness was recovered [21, 30–32]. We found that less than half (41.8%) of the initial MHO cohort maintained their MHO status (Figure 2), while in the initial MUO group, 71.3% maintained their MUO status, and 10.6% transitioned to the MHO phenotype (Figure 2). These transitions, as well as the baseline condition, may have an impact on health outcomes over time. Given the changeable nature of metabolic health, researchers have recently developed novel methodologies for the comprehensive assessment of metabolic health status by tracking its transitions [22, 23, 32, 33].

In the present analysis, we observed that AD risk highly depended on transitions of body mass and metabolic health phenotypes, as well as the baseline status. Specifically, maintenance of the MHO phenotype carried a significantly lower risk of AD development than maintenance of the MHNO phenotype (multivariable-adjusted HR, 0.62; 95% CI, 0.50–0.77). Among MUO subjects at baseline, those who transitioned to the MUNO had a higher risk of AD, and a transition to the MHO phenotype protected subjects from AD (multivariable-adjusted HR, 0.62; 95% CI, 0.50–0.78). The risk was even lower than that associated with a stable MHNO phenotype. Our analysis on the transition of metabolic health and obesity status is evidence of the protective effect of obesity *per se* against the risk of AD.

As weight loss has been identified as a preclinical indicator of AD [34–36], an inverse association between BMI and the risk of AD has been proposed [37, 38]. According to Suemoto et al., a higher fall in BMI over the first 4 years of the study was related with lower memory scores over the next decade, and poorer memory scores were related with a decrease in BMI, indicating reverse causation [39]. However, they did not take metabolic health into consideration; that is, the independent association of metabolic health with obesity was not evaluated. As dementia, including AD, occurs more frequently in metabolically unhealthy people, analyses are likely to represent the outcomes of metabolically unhealthy individuals if researchers do not consider metabolic health in their research protocols.

Our approach, considering the transition in obese metabolic health status, separately investigated the implication of obesity and weight loss on the risk of AD. In the present analysis, weight loss in the initial MUO group (i.e., MUO to MUNO transition) conferred a high risk of AD development (HR, 1.47; 95% CI, 1.30–1.66; Figure 3B), corroborating the previously demonstrated negative effect of weight loss. However, weight loss in the MHO group (i.e., MHO to MHNO transition) did not increase the risk of developing AD (HR, 0.79; 95% CI, 0.73–1.30; Figure 3B). These results imply that the weight loss, or the inverse association, cannot simply explain the relationship between high BMI and the risk of AD; the MHO phenotype seems to have a protective effect. We investigated the relationship between obesity and AD, considering the phenotypic transition as well as the impact of metabolic health in the obese people. Our approach thus could minimize the possibility of reverse causality.

There are many possible mechanisms explaining the protective effects of the MHO phenotype against the development of AD. Insulin-like growth factor I, which exerts neurotrophic effects on the hippocampus [40], could play a protective role against AD development. Takuya et al. reported that patients with AD had significantly lower IGF-1 concentration than controls without dementia in a case–control study [41]. Similarly, cross-sectional analyses from the Rancho Bernardo Cohort Study demonstrated higher IGF-1 levels to be associated with better cognitive test performance [42]. Significantly low plasma IGF-I has been observed in underweight individuals [43], which could at least partly explain the lower risk of AD in MHO subjects. Additionally, certain adipokines, such as leptin, secreted by adipose tissue may be another explanation [44]. Higher circulating levels of leptin have been associated with a reduced incidence of dementia and AD and with higher brain volume [45–47]. A recent study found that the MHO phenotype was positively associated with CSF-Aβ pathology, and this association remained significant after adjusting for other possible confounders [2], providing another mechanism potentially explaining the beneficial effects of MHO. Further studies are warranted to better understand the mechanisms and potential for interventions.

We observed that the MHO phenotype did not have a protective effect against vascular dementia in our additional analyses (Supplementary Tables 1 and 2). The pathogenesis of vascular dementia is likely associated with stroke, as vascular insufficiency is the primary pathophysiologic mechanism underlying both stroke and vascular dementia [48]. Previous studies have indicated that MHO subjects have a similar or slightly increased risk of stroke relative to MHNO subjects [23, 49–53]. As the pathogenesis of vascular dementia differs from that of AD, the effects of obesity on vascular dementia could also differ from the effects of obesity on AD, as suggested in our additional results, although the small number of events in this study cohort is insufficient for drawing concrete conclusions.

Our study had several limitations to be acknowledged. First, AD and other comorbid conditions were defined using claims data in the nationwide database. There might have been coding errors leading to overestimations or underestimations of disease prevalence. However, to improve diagnostic accuracy, we used both diagnoses based on ICD-10 codes and the prescription of medicines to define the diseases. Second, there was no information regarding apolipoprotein E4 phenotype, the nutritional status including vitamin levels, and thyroid function, all of which are known to be associated with AD [54–58]. Third, given the relatively short follow-up, this study may not have been adequately powered to fully assess interactions. Next, the criteria we used to define metabolic health and obesity were arbitrary. In our analysis, metabolically healthy (<2 risk characteristics) versus unhealthy were defined according to a modified National Cholesterol Education Program-Adult Treatment Panel III (NCEP-ATP III) definition of metabolic syndrome [59]. Since BMI was used as a marker of obesity in the analyses, waist circumference was not used in the definition of metabolic health, and a cutoff point of <2 risk factors was utilized instead of the traditional <3 risk factors to define “metabolically healthy.” In fact, several prior studies used the same definition of MHO as our present study for the same reasons [60, 61]. Additionally, since our data did not include serum insulin levels, we could not use HOMA-IR data to define metabolic health. Lastly, we tried to mitigate reverse causality between AD and body weight by assessing the transition; however, a short-term follow-up of 2 years might not be sufficient to completely rule out reverse causality. Further research with longer follow-up is warranted.

In summary, the present study illustrated different levels of AD risk according to metabolic health and obesity status in an elderly Asian population. The MHO phenotype was associated with a 27% lower risk of AD, and maintenance of the MHO phenotype further reduced the risk to a 38% risk reduction. Transition to the MHO phenotype from the MUO phenotype also carried a protective effect against AD development. Therefore, clinicians should counsel their obese patients about metabolic fitness to help prevent the development of AD.

## MATERIALS AND METHODS

### Study population

Data from the Korean National Health Insurance Service-National Health Screening Cohort (NHIS-HEALS), which included 514,866 people who had NHIS health screening checkups between 2002 and 2003. The entire structure and function of the Korean NHIS-HEALS are described in our Supplementary Materials and elsewhere [62]. During the index period of 2 years from January 1, 2009, through December 31, 2010, we assessed the demographic and biochemical data of the health screening cohort. This period was selected as the NHIS-HEALS has been documenting some biochemical measurements which are necessary for defining metabolic health, such as TG and HDL-C, since 2009 [62]. Additionally, the results from the next biannual examinations were used to assess serial changes in the obese and metabolic health status.

The subjects who died (*n* = 24,593) or had a history of dementia (*n* = 5,223) before or during the index period were excluded. Patients diagnosed with diseases that can lead to weight changes, either before their enrollment in the study inclusion or during follow-up, were excluded; people with malignancy (*n* = 37,212), thyroid disease (*n* = 19,120), head or spine trauma (*n* = 2,977), or previous major abdominal surgery (*n* = 2,824) were excluded. Individuals having missing values for the anthropometric or biochemical results were also excluded (*n* = 96,323). To determine the risk of AD according to late-life metabolic health and obesity, subjects aged ≥60 years were analyzed; people younger than 60 years were excluded (*n* = 189,747). Finally, 136,847 people were included in our analysis as a baseline study cohort. After the next biannual health examinations, 115,974 people were included in our analysis as a transition study cohort (Figure 1).

The institutional review board of Asan Medical Center approved this study (IRB No. 2020-0611). As this study analyzed the data from the NHIS-HEALS, which were fully anonymized and de-identified, informed consent from each participant was not obtained. This study was approved by the NHIS inquiry commission.

### Definitions of metabolic health and obesity states

According to their obese and metabolic healthy status, subjects were categorized into four groups: MHNO, MHO, MUNO, and MUO groups [59, 63]. Criteria used for the definition of obesity and metabolic health are described in our Supplementary Materials.

### Definitions of AD and metabolic comorbidities

AD was defined as at least two claims for AD (ICD-10 F00 or G30) with a prescription for anti-dementia drugs, such as rivastigmine, galantamine, memantine, and donepezil hydrochloride [19]. The study subjects were followed until the diagnosis of AD, death, or the end of the study period (December 31, 2015). Patients who were prescribed antidiabetic medications with diagnosis of type 2 diabetes, antihypertensive medications with diagnosis of hypertension and lipid-lowering medications with diagnosis of dyslipidemia were defined as having type 2 diabetes mellitus, hypertension or dyslipidemia, respectively. Definitions of AD and metabolic comorbidities in detail are provided in in our Supplementary Materials.

### Covariates

Covariates in the analyses included age, sex, income, smoking habits, alcohol consumption habits, and physical activity. Incomes were categorized as medical aid, lower half, and upper half; smoking habits as non-smoker or past/current smoker; alcohol consumption habits were categorized as none or drinker; and physical activity was categorized according to frequency per week (0, 1–4, or ≥5 times per week).

### Statistical analysis

Cox proportional hazards analyses were performed to estimate the hazard ratio (HR) and 95% confidence interval (CI) of AD. The risk of AD was assessed according to baseline metabolic health and obesity status, with the MHNO group as the reference. The risk of incident AD was further analyzed with consideration of the transitions in the metabolic healthy and obesity status, with reference to the stable MHNO group. All statistical analyses were performed using SAS Enterprise Guide software (version 7.1, SAS Institute, Inc., Cary, NC, USA). Detailed methods of statistical analysis are further described in our Supplementary Materials.

## Supplementary Materials

Supplementary Materials and Methods

Supplementary Tables
