# Supplementary material for: The risk of Alzheimer’s disease according to dynamic changes in metabolic health and obesity: a nationwide population-based cohort study
Source: Aging (Albany NY). 2021 Jul 8;13(13):16974–89. doi: 10.18632/aging.203255 (PMC8312469; doi:10.18632/aging.203255)
Supplement: Supplementary Materials and Methods [file aging-13-203255-s001.pdf]

SUPPLEMENTARY MATERIALS AND METHODS

Study population

Currently, the Korean National Health Insurance Service-National Health Screening Cohort (NHIS-HEALS) collects and manages databases on all healthcare services in Korea [62]. This cohort comprised a random sample of 514,866 individuals who represented approximately 10% of the source population, who underwent NHIS health screening examinations between 2002 and 2003, and who were followed until they were disqualified from health services due to death or emigration, or until the end of the study period in 2015 [62]. The data reported herein provide the findings from general health examinations of people who participated in biannual examinations.

Definitions of metabolic health and obesity states

Obesity (BMI ≥25 kg/m^2) and non-obesity (BMI <25 kg/m^2) were determined according to the Asia-Pacific criteria [63]; these definitions have been officially adopted by the Korean Centers for Disease Control and Prevention and the Korean Society for the Study of Obesity (KSSO) [64, 65]. Metabolic health was defined according to the Adult Treatment Panel III (ATP III) criteria as participants having none or one of the following risk factors [59]: (1) systolic BP ≥130 mmHg or diastolic BP ≥85 mmHg or taking antihypertensive medications; (2) TG ≥150 mg/dL or taking lipid-lowering medications; (3) FPG ≥100 mg/dL or taking antidiabetic medications; or (4) HDL-C <40 mg/dL in men and <50 mg/dL in women. Based on these criteria, all study participants were categorized into the following groups: (1) metabolically healthy, non-obese (MHNO), with BMI <25 kg/m^2 and ≤1 metabolic risk factor; (2) metabolically unhealthy, non-obese (MUNO), with BMI <25 kg/m^2 and ≥2 metabolic risk factors; (3) MHO, defined as BMI ≥25 kg/m^2 and ≤1 metabolic risk factor; and (4) MUO, with a BMI ≥25 kg/m^{2}and ≥2 metabolic risk factors.

Definitions of AD and metabolic comorbidities

To exclude the subjects who might have had dementia at the beginning of the study period, we used a broader definition than the definition for the outcome analysis; at least two claims for AD (ICD-10 F00 or G30), vascular dementia (ICD-10 F01.0, F01.1, F01.2, F01.3, F01.8, or F01.9), or other dementia (ICD-10 F02, F03, G23.1, G31.0, G31.1, G31.82, G31.83, G31.88, or F10.7), with a prescription for anti-dementia drugs such as rivastigmine, galantamine, memantine, and donepezil hydrochloride [19].

Patients who were prescribed antidiabetic drugs and with an ICD-10 code of E11 (noninsulin-dependent diabetes mellitus), E12 (malnutrition-related diabetes mellitus), E13 (other specified diabetes mellitus), or E14 (unspecified diabetes mellitus) were defined as having type 2 diabetes mellitus. Patients who were prescribed antihypertensive medications and with an ICD-10 code of I10 (essential [primary] hypertension), I11 (hypertensive heart disease), I12 (hypertensive CKD), I13 (hypertensive heart and CKD), or I15 (secondary hypertension) were defined as having hypertension. People who were prescribed lipid-lowering medications and with the ICD-10 code E78 (disorders of lipoprotein metabolism and other lipidemia) were defined as having dyslipidemia.

Statistical analysis

Continuous data are expressed as mean ± standard deviation (SD), and categorical data are expressed as percentages. Analysis of variance (ANOVA) and Scheffe's test for post hoc analysis or the chi-square test were used to compare the baseline characteristics of study participants based on their metabolic health and obesity status. We applied a multiple imputation procedure using a fully conditional specification method to impute missing values for smoking, alcohol consumption, and physical activity. The five imputed datasets were created with 20 burn-in iterations, analyzed by the same analytical procedures, and the results from these analyses were combined to obtain an overall estimate.

REFERENCES

64. Oh SW. Obesity and metabolic syndrome in Korea. Diabetes Metab J. 2011; 35:561–66. https://doi.org/10.4093/dmj.2011.35.6.561 PMID:22247896

65. Seo MH, Lee WY, Kim SS, Kang JH, Kang JH, Kim KK, Kim BY, Kim YH, Kim WJ, Kim EM, Kim HS, Shin YA, Shin HJ, et al. 2018 Korean Society for the Study of Obesity Guideline for the Management of Obesity in Korea. J Obes Metab Syndr. 2019; 28:40–45. https://doi.org/10.7570/jomes.2019.28.1.40 PMID:31089578
