# Supplementary material for: The risk of Alzheimer’s disease according to dynamic changes in metabolic health and obesity: a nationwide population-based cohort study
Source: Aging (Albany NY). 2021 Jul 8;13(13):16974–89. doi: 10.18632/aging.203255 (PMC8312469; doi:10.18632/aging.203255)
Supplement: Supplementary Tables [file aging-13-203255-s002.pdf]

SUPPLEMENTARY TABLES

Supplementary Table 1. Risk of vascular dementia according to baseline metabolic health and obesity status.

Baseline category<br>BMI<br>Metabolic health status	MHNO<br><25 kg/m^2<br>0–1 risk factors	MHO<br>≥25 kg/m^2<br>0–1 risk factors	MUNO<br><25 kg/m^2<br>≥2 risk factors	MUO<br>≥25 kg/m^2<br>≥2 risk factors
n (% of total)	33, 049 (17.4)	10, 445 (5.5)	53, 958 (28.4)	39, 395 (20.8)
Number of events (%)	232 (0.70)	66 (0.63)	534 (0.99)	332 (0.84)
Crude HR (95% CI)	1 (ref)	0.89 (0.68–1.17)	1.43 (1.22–1.67)	1.19 (1.01–1.41)
Multivariable-adjusted HR (95% CI)[a]	1 (ref)	0.87 (0.67–1.15)	1.41 (1.21–1.64)	1.17 (0.99–1.38)

[a]Adjusted for baseline age, sex, income, smoking, alcohol drinking, and physical activities.
Abbreviations: MHO: stable metabolically healthy obesity; MUO: metabolically unhealthy obesity; MUNO: metabolically unhealthy obesity; MHNO: metabolically healthy non-obesity.

Supplementary Table 2. Risk of vascular dementia according to transitions of metabolic health and obesity status.

Baseline category	MHNO			
Follow-up category	MHNO	MHO	MUNO	MUO
n (% of baseline category)	17, 812 (63.0)	994 (3.5)	8, 590 (30.4)	866 (3.1)
Number of events (%)	83 (0.47)	6 (0.60)	76 (0.88)	8 (0.92)
Crude HR (95% CI)	1 (ref)	1.29 (0.56–2.94)	1.92 (1.41–2.62)	1.97 (0.96–4.08)
Multivariable-adjusted HR (95% CI)[a]	1 (ref)	1.28 (0.56–2.93)	1.89 (1.39–2.58)	1.95 (0.95–4.04)
Baseline category	MHO			
Follow-up category	MHNO	MHO	MUNO	MUO
n (% of baseline category)	1, 095 (12.1)	3, 780 (41.8)	653 (7.2)	3, 514 (38.9)
Number of events (%)	4 (0.37)	15 (0.40)	2 (0.31)	17 (0.48)
Crude HR (95% CI)	0.78 (0.28–2.12)	0.84 (0.49–1.46)	0.66 (0.16–2.67)	1.03 (0.61–1.74)
Multivariable-adjusted HR (95% CI)[a]	0.77 (0.28–2.10)	0.84 (0.48–1.45)	0.65 (0.16–2.64)	1.01 (0.60–1.70)
Baseline category	MUNO			
Follow-up category	MHNO	MHO	MUNO	MUO
N (% of baseline category)	9, 211 (20.4)	623 (1.4)	31, 657 (70.1)	3, 665 (8.1)
Number of events (%)	55 (0.60)	5 (0.80)	277 (0.88)	30 (0.82)
Crude HR (95% CI)	1.28 (0.91–1.80)	1.72 (0.70–4.24)	1.90 (1.49–2.43)	1.78 (1.17–2.70)
Multivariable-adjusted HR (95% CI)[a]	1.26 (0.90–1.78)	1.69 (0.69–4.18)	1.88 (1.47–2.40)	1.74 (1.14–2.64)
Baseline category	MUO			
Follow-up category	MHNO	MHO	MUNO	MUO
n (% of baseline category)	1, 039 (3.1)	3, 568 (10.6)	4, 797 (14.3)	24, 110 (71.9)
Number of events (%)	6 (0.58)	19 (0.53)	46 (0.96)	153 (0.63)
Crude HR (95% CI)	1.23 (0.54–2.83)	1.12 (0.68–1.85)	2.07 (1.44–2.96)	1.36 (1.04–1.78)
Multivariable-adjusted HR (95% CI)[a]	1.22 (0.53–2.79)	1.11 (0.67–1.83)	2.02 (1.41–2.90)	1.33 (1.02–1.74)

[a]Adjusted for baseline age, sex, income, smoking, alcohol drinking, and physical activities.
Abbreviations: MHO: stable metabolically healthy obesity; MUO: metabolically unhealthy obesity; MUNO: metabolically unhealthy obesity; MHNO: metabolically healthy non-obesity.
